# Exploring Intrapersonal Determinants of Older Adults’ Group Exercise Engagement in Taiwan: A Theory of Triadic Influence Perspective

**DOI:** 10.1007/s10823-025-09547-8

**Published:** 2025-10-25

**Authors:** Yi-Chieh Yeh, Wen-Yu Chiu

**Affiliations:** National Taiwan University of Sport, Taichung, Taiwan

**Keywords:** Regular exercise, Physical activity, Successful ageing, Public health

## Abstract

As global population ageing accelerates, sustaining engagement in health-related activities has become a key strategy to promote healthy ageing and maintain quality of life in Taiwan. To address this issue, the present study draws on an established theoretical framework to offer a culturally sensitive and practice-oriented interpretation of regular health-promoting behaviours, thereby informing actionable strategies for health practitioners. This qualitative case study, grounded in the Theory of Triadic Influence, examined perspectives on the determinants that shape older adults’ long-term engagement in group-based exercise. We conducted individual interviews with five participants who had consistently attended group exercise programme for over five years. By focusing on six personal determinants: personality, sense of self, locus of control, self-determination, general skills, and self-efficacy, we explored the commonalities that caused or explained those older adults’ sustained participation. The findings revealed several shared factors, including self-directed motivation, internalised agency, and awareness of physical decline, which collectively supported sustained engagement in health-promoting behaviours. In contrast, personality traits, self-concept, and perceived control exhibited considerable diversity among participants. The study further offered culturally grounded interpretations of these divergent perspectives, highlighting how individual values and motivations were shaped by sociocultural contexts. This study highlights the need for practitioners to consider diverse personality traits and emotional sensitivities, rather than assuming all participants are extroverted. Supporting individual progress through clear guidance can enhance self-efficacy and foster continued participation. Emphasising small personal achievements over immediate performance may help reduce self-doubt and prevent disengagement.

## Introduction

The global population is experiencing a demographic shift towards ageing. Since 2017, individuals aged ≥ 60 years have accounted for one in eight of the global population (World Health Organization, 2020). According to official data from the National Development Council of Taiwan (2024), Japan, France, and Germany have already transitioned into super-aged societies, and Taiwan is expected to reach this demographic milestone within the next year, meaning that at least one in five individuals will be aged 65 or older. This ageing demographic and declining birth rate have intensified caregiving pressures, as the responsibility for supporting older parents has traditionally fallen on adult children. Within this cultural context, the Confucian ethic of filial piety remains highly valued (Yeh et al., 2013), yet it is increasingly strained as shrinking family sizes and weakening intergenerational networks make family-based elder care more difficult to sustain. Thus, promoting healthy longevity among older adults is increasingly recognised as a public health priority, aimed at enhancing quality of life and easing the burden on the older adult populations.

The notion of ‘health’ was once defined not merely as the absence of infirmity or disease but as a state of complete physical, mental, and social well-being (World Health Organization, 1946). Numerous researchers have endeavoured to assess the effectiveness of exercise in improving physical functioning among older adults (Burke et al., 2010; Irez et al., 2011; Roma et al., 2013). In addition, various studies have shown that optimal mobility can positively influence older adults’ psychological benefits by promoting mental wellness and self-esteem as well as reducing loneliness and social isolation (Hwang et al., 2018). Group exercise is recognised as an effective intervention to enhance social engagement in older adults (Cohen-Mansfield et al., 2015; Cyarto et al., 2008; Gardiner et al., 2018; Stuart et al., 2009).

To inform intervention strategies, contemporary empirical studies (Leung et al., 2021; Royse et al., 2023) have adopted the social-ecological model as a structural framework to identify factors that both motivate (Bauman et al., 2012; Dionigi, 2007; van Stralen et al., 2009) and hinder older adults’ participation in group exercise programmes. Identified barriers include fear of injury, lack of time and motivation, social constraints, limited financial support, and restricted accessibility (Baert et al., 2011; Bethancourt et al., 2014; Hamer et al., 2021; Maula et al., 2019). Despite studies adopting the social-ecological model have provided compelling evidence regarding the factors that influence participation, the model remains largely descriptive and does not sufficiently address how these determinants interact within individuals to shape long-term behavioural engagement, particularly from an intrapersonal perspective.

### The Theory of Triadic Influence

**Fig. 1:**
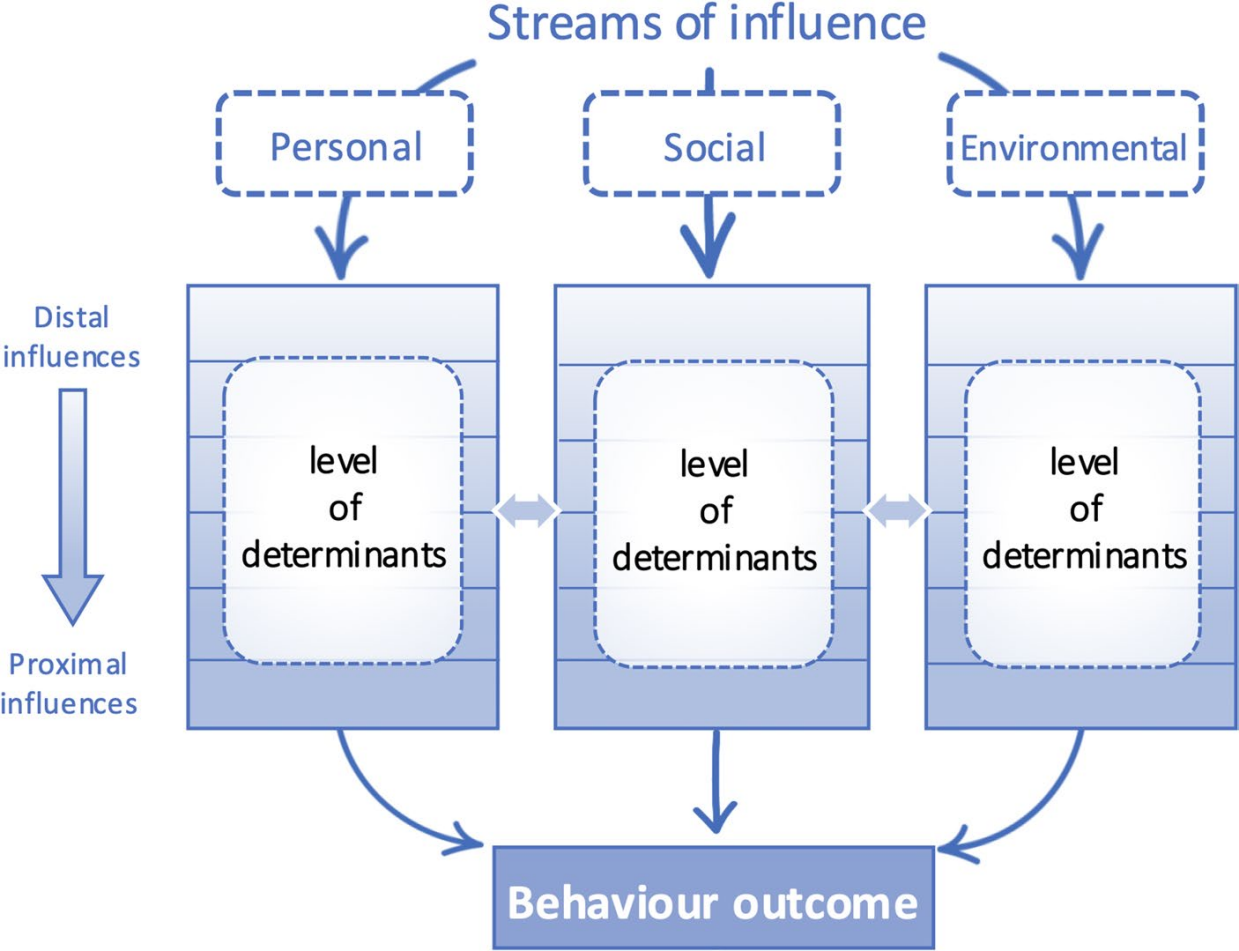
Conceptual diagram of the three streams and levels of behavioural influence adapted from Flay et al. (2009)

In light of the growing imperative to sustain older adults’ engagement in physical activity, a deeper psychological understanding of intrapersonal determinants is essential to inform the design of effective interventions. Accordingly, this study is grounded in the Theory of Triadic Influence (TTI), which not only identifies the interwoven factors underlying specific behaviours but also explains how multiple determinants shape behavioural outcomes through structured, hierarchical pathways. Specifically, the model offers a comprehensive overview that integrates multiple influences within a two-dimensional matrix defined by the streams of influence and the levels of causation. The three streams of personal, social, and environmental influence, each contains determinants operating at hierarchical levels from distal to proximal (Fig. 1).

Within one of the streams of influence—personal stream—the starting point is the distal level, which consists of relatively stable biological traits (e.g., personality). According to the model, this higher-level determinant directly shapes lower-level social factors (e.g., sense of self) and personal factors (e.g., locus of control). In turn, the next tier is proposed to influence more behaviour-specific constructs, including internal determinations (e.g., self-determination) and general competencies (e.g., general skills), both of which are essential for a person to carry out a particular behaviour. Ultimately, the TTI suggests that these tiers of determinants progressively lead to a person’s confidence (e.g., self-efficacy) regarding a specific behaviour (Flay et al., 2009). These levels are linked through a probabilistic causal chain, whereby higher-level determinants progressively shape those at lower levels, thereby increasing the likelihood of specific behavioural outcomes (Flay & Petraitis, 1994; Flay et al., 2009; Snyder & Flay, 2012). Therefore, the TTI serves as a strategic and practical blueprint by presenting feasible approaches that enable health promotion practitioners to design multi-level interventions that are both conceptually coherent and operationally efficient.

### Study Aim

This study focused on the personal stream of influence within the TTI framework because it constitutes the most direct and central influence on behaviour. Furthermore, qualitative inquiry is particularly well suited to exploring the inner structural pathway underpinned by the six determinants of the personal stream. Accordingly, this study aims to explore how older adults perceive and experience the six personal determinants that collectively shape and motivate their sustained participation in group exercise programmes.

## Methods

### Research Design

A case study approach was adopted to gain an in-depth understanding of the determinants influencing health-related behaviours at the individual level. This single-case study design followed Yin’s (2003) theoretical propositions strategy and was grounded in the TTI framework, enhancing data consistency and ensuring a clear justification of findings. This study employed in-depth individual interviews with adults at a life stage often marked by profound transitions, such as retirement, evolving health concerns, and shifts in life purpose. Drawing on their valuable personal reflections and experiences, the study specifically focused on individuals who had maintained long-term participation in a group-based exercise programme, with the aim of exploring how their perceptions of the determinants supported sustained engagement. Data were analysed using a categorisation strategy (Maxwell, 2012; McMillan & Schumacher, 2001), with the TTI framework serving as a structured lens for interpretation and development of compelling analytic conclusions. Ethical approval was granted by [redacted] Institutional Review Board.

### Participants

Case study research aims to explore the complexities of a defined entity or case (Yin, 1994). In this study, all five participants were recruited from a pool of 24 individuals enrolled in the 2024 fall semester class of the same programme, which included seven types of exercise: yoga, resistance training, stretching, gymnastics, Qigong, Petanque, and orienteering. The inclusion criteria were as follows: participants had to be 60 years of age or older. They were also required to have been continuously enrolled in a programme (not necessarily offered by the same university or college) for at least five consecutive years, with an attendance rate of 80% or higher. Finally, participants had to be free from physical injuries or mental health conditions that could hinder participation. The final case selection included two women and three men, aged 64–69 years (mean age 65 years; see Table 1). All participants provided written informed consent prior to the interviews. The study procedures, including its purpose, confidentiality measures and the use of audio recordings, were verbally explained in full detail before consent was signed. Eligibility was also confirmed at this stage. No minors were involved in this study.

**Table 1:** Participant characteristics

Participant code	Age	Sex	Consecutive enrolment (year)	Marital status	Previous occupation	Education level
P1	65	Male	8	Married	Mechanical designer	College diploma
P2	67	Male	12	Never married	Sales executive	High school
P3	64	Female	6	Married	Employee in a trading company	Bachelor’s degree
P4	64	Male	6	Married	Employee in a trading company	Bachelor’s degree
P5	69	Female	8	Married	public servant	High school

*All five participants were enrolled in the 2024 fall semester class of the same programme, which included seven types of exercise: yoga, resistance training, stretching, gymnastics, Qigong, Petanque, and orienteering.

### Data Collection

This study received ethical approval from the Institutional Review Board of a university in Taiwan and was conducted in accordance with the principles of the Declaration of Helsinki. Participants were first informed about the study, including that each individual face-to-face interview would last approximately 60 min and that the process would be audio-recorded. The informed consent form was then distributed. Those who expressed willingness to participate were asked to sign the form. Once consent was obtained, the researcher arranged the interviews individually. All interviews were conducted in the same classroom at a national university, and recruitment was carried out through an exercise programme organized by the same university. To ensure confidentiality, each participant was assigned a coded ID, and all transcripts were de-identified.

All interviews were scheduled over the course of one week and conducted face-to-face by the first author in Mandarin, and audio-recorded with participants’ consent. A semi-structured format was employed, guided by an interview protocol with open-ended questions designed to elicit participants’ personal experiences and perspectives. An interview guide was developed based on the six determinants of the personal stream of influence in the TTI framework. To ensure conceptual clarity and avoid arbitrary definitions, these determinants were anchored in established theoretical constructs: personality traits—the Big Five (Flay et al., 2009), sense of self (Flury & Ickes, 2007), locus of control (Rotter, 1966), self-determination and general skills (Snyder, 1991), and self-efficacy (Bandura, 1977). The interview guide consisted of multiple sections (see Appendix), each containing several prompt questions developed around six determinants, which are referred to as topics in this study. The final version of the guide was reviewed and confirmed by researchers with recognized expertise in qualitative research. Based on the participants’ responses, targeted probing questions were posed to encourage open communication and further clarification.

### Group-Based Exercise Programme

The Taiwan Ministry of Education (MOE) is a national institution overseeing the education system and formulating policies, curricula and regulations to enhance educational quality and foster innovation. Since 2008, following a 2006 policy initiative, the MOE has funded exercise programmes organized by higher education institutions. Each university or college is eligible to host one programme providing adults aged 55 and above with opportunities to participate in structured exercise courses. These programmes operate on an academic-year basis, offering five to seven types of exercise classes each week at fixed times, with each semester lasting 12 to 14 weeks (Ministry of Education, n.d.). This registration-based programme has launched several forms of exercise activities, including yoga, Petanque, Qigong, resistance training, aerobics, gymnastics, and Taiko drumming, with each class lasting 60 min. Each class consisted of at least 20 participants.

### Data Analysis and Rigor

Interviews were audio-recorded and transcribed verbatim within 12 h of each interview to ensure accuracy and maintain data integrity. In this case study, a categorisation strategy was employed to interpret the data (Merriam, 1988; Weiss, 1994). In line with the TTI framework, six determinants from the personal stream of influence: personality, sense of self, locus of control, self-determination, general skills, and self-efficacy, were used as the main topics for analysis. The analysis was conducted manually using a categorisation strategy (Maxwell, 2012; McMillan & Schumacher, 2001). The first author read all transcriptions and coded meaning units through an iterative process and then sorted the coded segments under their corresponding topics. Descriptive themes were generated from the coded units as ‘substantive categories.’ Based on these substantive categories, abstract concepts were derived to establish the underlying meanings of each topic, forming ‘organised categories’ (Table 2). Once the manifest content was generated, it was reviewed by three scholars with expertise in qualitative research and the TTI framework. With their critical feedback and professional insights, the content was refined and finalised through discussion. Translation was carried out by the first author, who was familiar with the interview setting and circumstances. A peer-checking process was then conducted, in which the translated transcripts were presented to the other experienced researcher who reviewed a subset of the translations against the original Mandarin text to ensure contextual and cultural appropriateness. Any discrepancies were discussed and resolved through consensus.

**Table 2:** Analysis process examples

Meaning unit	Substantive category	Organised category	Topic
*Sure*,* luck plays a role in life. But if you don’t have the experience*,* and you haven’t put in the work*,* it doesn’t count for much. We spend our whole lives from youth to retirement gaining experience*,* and that’s how we grow.*	Sense of control over life outcomes	Belief in internal control	Locus of control
*If you don’t put in some effort or make some kind of sacrifice*,* how can you expect good results? You have to work hard if you want something good to come out of it.*	Persistence and personal sacrifice for achievement		
*I guess I’m more of a fatalist. When it comes to handling things*,* I tend to just… go with the flow. I feel like things work out the way they’re meant to*,* so why push yourself to the edge? If something’s meant to be mine*,* it will be. If it’s not*,* no matter how hard you try… maybe it just wasn’t part of the plan.*	Acceptance of uncontrollable outcomes	Belief in external control	

This study used two general approaches to establish data credibility (Cho & Trent, 2006). Member checking was employed as a transactional validity technique to enhance data accuracy and establish consensus. Each participant was provided with a form containing selected transcript excerpts along with the corresponding categorised themes and concepts, and was asked to review these independently. All participants confirmed that the transcript excerpts accurately reflected their accounts and that the thematic categorisation was appropriate; no adjustments were requested. The transformational approach is a reflexive process in which the researcher critically engages with the inquiry, participants, and evolving interpretations to strengthen validity and deepen understanding. To this end, targeted probing questions were consistently used during the interviews to refine any ambiguities in understanding based on prior responses.

## Results

**Fig. 2:**
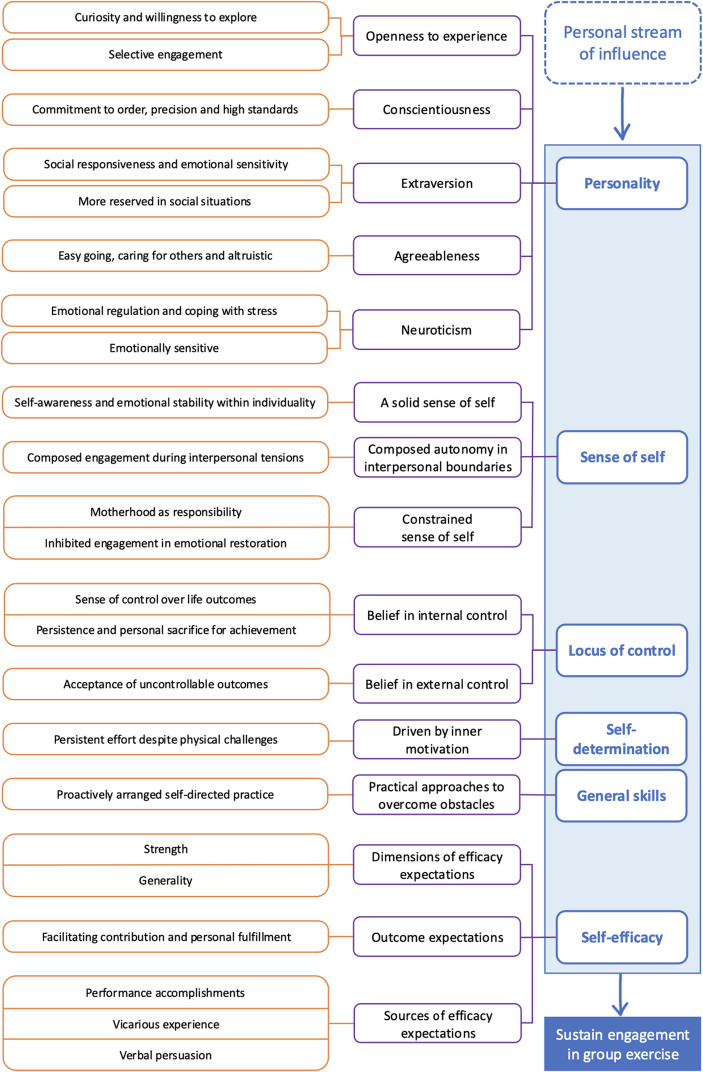
Overall substantive and organised categories

The analysis of the six determinants was conducted through participants’ narratives, from which overarching insights were identified and marked in orange as substantive categories. Higher-order concepts were subsequently abstracted and marked in purple as organised categories. These categories were visually mapped to illustrate the distinctive characteristics of each determinant (Fig. 2).

While the first three determinants revealed heterogeneous perspectives, with participants differing in personality expressions, self-construal, and orientations of control, the last three highlighted more consistent commonalities in sustaining participation. Across narratives, self-determination was evident as participants acknowledged age-related limitations and reframed the pursuit of perfect performance into the goal of giving their best effort. This mindset reinforced motivation and manifested in persistent attendance, deriving satisfaction from consistent effort, and ultimately transforming participation into a long-term commitment. Complementing this, general skills were demonstrated through practical approaches to overcoming obstacles, with participants maintaining attendance despite physical challenges and, in some cases, proactively arranging self-directed practice outside of class to enhance performance. Building on these, self-efficacy emerged through reinforced confidence in the ability to adapt to challenges and improve physical conditioning, creating a positive feedback loop in which accomplishments fostered a sense of fulfilment that sustained engagement.

### Personality

Participants’ personality-related experiences reflected the key traits of openness, conscientiousness, extraversion, agreeableness, and neuroticism.

#### Openness to Experience

Two participants demonstrated an attitude of embracing new experiences in their general lives, regardless of their age. Their openness reflected a proactive stance towards growth and change, extending beyond exercise participation into a broader orientation toward life. In contrast, one participant adopted a more selective approach, engaging only when he had carefully weighed the activity and judged it to be meaningful or beneficial. His participation reflected prudent evaluation in everyday life choices rather than a spontaneous openness to new experiences.

*Curiosity and willingness to explore:* Participants (P3 and P4) expressed a sustained openness to unfamiliar experiences. This was particularly evident in their enthusiasm for seizing new opportunities. They resisted the notion that ageing should serve as a barrier to continued exploration and engagement in life.

*I’ve always been a curious one…basically like a Curious George. As long as it’s not something dangerous, I’m open to trying anything. I think staying open-minded keeps you young. You’ve gotta keep that sense of curiosity. Some people give up too quickly, like saying, ‘Oh, never mind, I won’t try it.’ That’s not me. (P3, Female)*

*Selective engagement:* One participant approached new experiences with a cautious and deliberative mindset. His engagement was more consistent with the situational approach, often choosing to participate only when he felt it was necessary.

*Some people say I seem impassive in group settings, like I’m not really that involved. But honestly, it’s not that I avoid participating. I just feel that if there’s a real reason to be involved, then I’ll definitely join in. If not, I won’t go out of my way to be actively engaged. (P2, Male)*

#### Conscientiousness

All five participants exhibited a common personality trait characterised by a strong sense of duty and pursuit of perfection, as reflected in their high personal standards and meticulous attention to detail in everyday tasks.

*Commitment to order, precision and high standards:* The participants expressed a strong sense of responsibility and clear drive for excellence. Their pursuit of precision and order reflected not only a desire to do things right but also a deeper commitment to self-discipline and personal accountability.

*The way I do things, I tend to give it my all and try to think things through as carefully and thoroughly as I can. (P1, Male)*

#### Extraversion

The findings indicate that one participant showed a distinctly extroverted disposition. In contrast, the remaining four participants were not inclined to present themselves assertively or engage frequently with unfamiliar individuals.

*Social responsiveness and emotional sensitivity:* Among the five participants, only one demonstrated a more extroverted personality, marked by active social engagement and sensitive awareness of others’ emotional needs.

*I quite enjoy chatting with people. There was this older lady in our group, …more than ten years older than me …, and she was always so polite. She would usually sit there quietly by herself. It’s not that she was unfriendly, just more on the reserved side. So I would go and talk to her. (P3, Female)*

*More reserved in social situations:* The remaining participants tended to maintain a low profile in the group settings and demonstrated a relatively limited inclination to interact with strangers. Although not overtly introverted, they were more socially engaged in familiar contexts or when interactions were deemed necessary.

*I’m not really the type who would go out of my way to start a conversation with strangers. I’m not exactly outgoing. But if it’s someone I know, I’ll talk more; if not, I tend to keep quiet. It really depends on the vibe of the situation. (P4, Male)*

#### Agreeableness

All the participants demonstrated relational sensitivity in their social interactions and balanced emotional connections with context-dependent engagement.

*Easy going, caring for others and altruistic:* The participants tended to withhold strong personal opinions to preserve social harmony during their interactions. They valued emotional connections while maintaining respectful boundaries and adjusting their level of engagement based on social context.

*I’m willing to stay and connect with people, to build relationships..., and I think caring about others can make you happy. Of course, that doesn’t mean getting involved in their personal business... Sometimes, through those conversations, they are able to open up and share things about themselves... That feels meaningful to me. When it comes to interacting with others..., I think the most important thing is to be genuine. (P3, Female)*

#### Neuroticism

Three of the five participants demonstrated emotional stability and ability to regulate their emotions in response to external stimuli. However, one participant noted that her emotional reactions were triggered more easily during interactions with family members.

*Emotional regulation and coping with stress:* Three of the participants (P1, P2, and P3) exhibited emotional stability and self-regulation. When facing stress, they believed that excessive worry was unhelpful.

*These days, I don’t get easily triggered or …react strongly to stress. I suppose it’s because, through a lifetime of experience, you come to realise that worrying too much doesn’t help. Sometimes, you just don’t have the strength of mind for that. So really, what’s the point? (P3, Female)*

*Emotionally sensitive:* One participant acknowledged that she was emotionally affected by family dynamics, particularly during interactions with close family members.

*I can be pretty sensitive, honestly. When it comes to interactions with my family, I sometimes get caught up in emotions, and feel upset for a couple of days. (P5, Female)*

### Sense of Self

The interview analysis identified three categories related to participants' self-understanding. These included internal awareness, responses to social interactions, and factors that appeared to limit self-perception.

#### A Solid Sense of Self

Four participants were attuned to their emotional states and personal values, and responded to external pressures with calm reasoning and emotional self-regulation.

*Self-awareness and emotional stability within individuality:* The participants (P1, P2, and P3) demonstrated a clear sense of self-awareness, including an understanding of their emotional responses and personality traits. They were able to regulate their emotions appropriately while maintaining personal principles in group settings. Although they valued social harmony, they emphasised autonomy and did not compromise their individuality to please others.

*I’m friendly to most people. But if it means... pleasing others at the cost of sacrificing myself, then I’m not going to do it. I still insist on being true to who I am. Maybe some people talk badly about me behind my back, but these days, I don’t really care anymore. (P3, Female)*

#### Composed Autonomy in Interpersonal Boundaries

Two participants maintained a respectful distance in their relationships, remained true to their principles, and responded to criticism with quiet flexibility. This reflects the steady balance between self-assurance and social awareness.

*Composed engagement during interpersonal tensions:* Participants (P3 and P4) were open to engaging in critical feedback, but tended to disregard non-constructive criticism without confrontation.

*When there’s some kind of negative comment about me… well, if it’s from someone I’m close to, I’ll take it in and reflect on it. But if it’s from someone I don’t really know, I just let it slide with a smile. I’m not the type to get into confrontations, especially with someone I’m not close to. (P4, Male)*

#### Constrained Sense of Self

While four participants demonstrated clarity in their sense of self, this study also uncovered circumstances in which self-perception appeared limited or restrained. In particular, one participant’s account highlighted subtle internal barriers. These reflections are organised into two distinct categories.

*Motherhood as responsibility:* The participant’s narrative reflected a motherhood mindset deeply rooted in traditional family norms, in which a mother is expected to place her family’s needs before her own. This cultural expectation might impose disproportionate responsibilities on her, making the concepts of self-care and self-love feel distant and unfamiliar.

*I don’t really understand myself, and I guess I’ve always been passive about that. People talk about loving yourself, but I wonder, what does that even mean? For me, everything has always centred around my family, and I always put them first…. That’s my responsibility. Since I’m a housewife, that’s the role I’m supposed to play. (P5, Female)*

*Inhibited engagement in emotional restoration:* The participant also described how she had been conditioned by her upbringing to stay home during her free time rather than seeking solitude or emotional renewal outside, even though restorative and mood-lifting activities were often encouraged.

*Even when I’m in a bad mood, there’s nothing I can really do about it. Life still goes on, right? So I just stay at home and feel upset for a while. I guess it’s the way we were raised in traditional families. We’re not used to going out with friends. Of course, there are occasional get-togethers, but going out alone to relax almost never happens for me. I don’t really go looking for alone time or anything like that. If I have free time, I just stay at home. (P5, Female)*

### Locus of Control

#### Belief in Internal Control

Four out of five participants emphasised that their own efforts and abilities were essential to success, rather than relying on external circumstances, which they viewed as unstable and unreliable. They tend to trust their capabilities to influence outcomes through personal control.

*Sense of control over life outcomes:* Participants (P1, P4, and P5) emphasised that life outcomes were primarily the result of personal ability rather than external circumstances or luck. They viewed the accumulation of experience, built steadily from youth, as the foundation for growth and achievement.

*Sure, luck plays a role in life. But if you don’t have the experience, and you haven’t put in the work, it doesn’t count for much. We spend our whole lives from youth to retirement gaining experience, and that’s how we grow. (P5, Female)*

*Persistence and personal sacrifice for achievement:* Beyond attributing positive outcomes to personal experiences and abilities, one participant also emphasised that achieving desirable results required wholehearted commitment and persistent effort.

*If you don’t put in some effort or make some kind of sacrifice, how can you expect good results? You have to work hard if you want something good to come out of it. (P5, Female)*

#### Belief in External Control

In contrast, one participant expressed the belief that life outcomes are primarily determined by fate rather than by her own efforts or abilities.

*Acceptance of uncontrollable outcomes: *The participant expressed the belief that life unfolds according to predetermined paths, preferring to accept destiny and believing that effort alone cannot alter what is to happen.

*I guess I’m more of a fatalist. When it comes to handling things, I tend to just... go with the flow. I feel like things work out the way they’re meant to, so why push yourself to the edge? If something’s meant to be mine, it will be. If it’s not, no matter how hard you try... maybe it just wasn’t part of the plan. (P3, Female)*

### Self-Determination

#### Driven by Inner Motivation

Participants generally demonstrated an awareness of their physical abilities and recognised the gradual decline in their overall physical condition that comes with ageing. They did not push themselves beyond their limits but accepted their physical constraints, believing that doing their best was fulfilling. Nevertheless, they maintained a persistent attitude, striving to keep up with their classes as much as possible.

*Persistent effort despite physical challenges:* When encountering age-related physical limitations, participants (P1, P4 and P5) encouraged themselves to keep practising while adjusting their expectations. Rather than giving up, they accepted their current capacities and continued striving towards their goals by doing their best.

*Our bodies are stiff now, so we just can’t stretch the way the instructor does. But she always encourages us to do our best,... and we just try as much as we can. (P5, Female)*

### General Skills

#### Practical Approaches to Overcome Obstacles

Despite challenges during exercise, the participants maintained consistent participation. Two also proactively arranged extra sessions outside of class, taking practical actions to improve performance.

*Proactively arranged self-directed practice:* When encountering physically demanding movements in class, two participants (P3 and P4) deliberately arranged additional practise opportunities to overcome obstacles and achieve their exercise goals.

*If the class feels too hard and I start falling behind, I’ll make time to practise on my own, just so I can keep up in the next sessions. (P4, Male)*

### Self-Efficacy

#### Dimensions of Efficacy Expectations

The beliefs about future engagement in group exercises reflected two dimensions of efficacy expectation: strength and generality.

*Strength:* All five participants expressed a strong and enduring sense of confidence in their ability to continue participating in the exercise programme. They conveyed firm determination and clear anticipation of future involvement.

*I'll keep participating, yah, unless there's a health problem. Otherwise, I don’t think I'd stop. (P5, Female)*

*Generality:*One participant also expressed confidence in his ability to start weight training, suggesting a belief in his physical capabilities that could be applied across various contexts.

*I’ve been feeling more and more confident in myself. In the future, I’d like to challenge myself...like getting into strength training. I actually feel pretty confident about that, since the harder you train, the more progress you’ll see. (P1, Male)*

#### Outcome Expectations

The findings indicated that participants’ evaluations of outcomes related to their health behaviours reflected a combination of social recognition and a sense of personal value derived from helping others.

*Facilitating contribution and personal fulfilment:* Through regular exercise, individuals developed better physical health, which enabled them (P2, P3, and P4) to support others and in turn fostered a stronger sense of self-worth.

*If you don’t have your health, you can’t even take care of yourself. You’d be spending all your time going back and forth to the hospital. How would you have time to do anything else? But if you join this group activity and keep exercising here, your chronic pain eases up. Then you’ll have the ability to help others. (P2, Male)*

#### Sources of Efficacy Expectations

Based on the accounts of these five participants, this study identified three key sources of efficacy expectations: performance accomplishments, vicarious experiences, and verbal persuasion.

*Performance accomplishments:* Reflecting on their past health conditions and physical performance, participants (P1, P2, and P3) reported noticeable improvements over time after engaging in regular exercise. These experiences signified physical progress, which, in turn, enhanced their confidence and reinforced continued participation.

*Back then, my immune system wasn’t functioning properly, and the doctor told me that if I relied only on medication, the dosage would just keep increasing. …so I decided to join this exercise programme. After about a year, I noticed that my symptoms weren’t as noticeable anymore. I even started forgetting to take my meds sometimes, you know? …The discomfort in my body had eased up too. (P2, Male)*

*Vicarious experience:* Four participants (P1, P2, P4, and P5) expressed awareness of the common phenomenon of prolonged bedridden years among older adults in Taiwan. This realisation appeared to strengthen their commitment to engaging more intentionally in managing their health. They identified the value of regular preventive exercise as a meaningful way to maintain physical function, while preserving a sense of dignity and autonomy in later life.

*If your health is good, your quality of life will get better too. These days, people often say the shorter your bedridden years are, the better. I mean, what’s the point of living into your eighties or nineties if you end up spending decades stuck in bed? That’d be pretty miserable, haha. (P1, Male)*

*Verbal persuasion:* Professional guidance from instructors supported the participants’ engagement. They (P4 and P5) believed that expert instruction helped them improve and master the proper exercise techniques. Instructors’ empathetic and encouraging teaching styles also enhanced their confidence and strengthened their willingness to stay engaged.

*Having professional instructors guiding us has been a huge help, whether it’s for developing our technique, deepening our academic knowledge, or improving our overall health. (P4, Male)*

## Discussion

This study explored how older adults in Taiwan perceive and identify the key determinants contributing to their long-term engagement in group exercises. Participants’ diverse perspectives were initially categorised, then further interpreted through a cultural lens to capture context-specific meanings and values.

### Personality

Research on personality traits is well established in multiple fields, and their influence on health behaviours has received considerable empirical attention (Hall et al., 2014; MacCann et al., 2015; Siegler et al., 2013). Accordingly, the present study draws on the Big Five theoretical framework to define the construct of personality. Higher levels of openness to experience have been associated with better maintenance of physical functioning in older adults (Martin et al., 2006). Interestingly, our findings indicate that participants with the longest continuous attendance in the group exercise programme exhibited lower levels of openness to experience. This trait, typically characterised by a preference for routine, familiarity, and a conservative, practical outlook, may help explain the divergence from prior studies. It is possible that previous participation in the programme fostered a sense of continuity and routine, thereby supporting sustained engagement regardless of trait openness. A systematic review highlighted the associations between personality traits and successful ageing, reporting that higher levels of conscientiousness and extraversion were positively associated with successful ageing, whereas neuroticism was negatively associated (Pocnet et al., 2021). Moreover, the study highlighted the significant direct effect of personality traits on health outcomes. In contrast to previous research, this study identified four participants with low levels of extraversion and one participant with high levels of neuroticism. This apparent contradiction may be explained through the framework of the TTI, which conceptualises personality as the most distal determinant of behaviour within the personal stream (Flay et al., 2009). Consequently, its influence may be moderated by the proximal factors. Finally, consistent with our findings, a longitudinal study by Lodi-Smith and Roberts (2012) reported that older adults aged 60 to 86 years who scored higher in agreeableness demonstrated greater social engagement.

### Sense of Self

Participants exhibited clear self-awareness and demonstrated the capacity to regulate dynamic interactions between external influences and internal cognitive processes. However, one female participant struggled to construct a coherent sense of self, as her self-perception appeared to be shaped and constrained by social expectations. Across generations, women’s roles have been inherited through cultural transmission. In traditional Chinese culture, a woman is expected to marry and fulfill societal expectations of reproduction. As a virtuous wife, she is typically associated with responsibilities such as domestic work, childcare, and even the performance of emotional labor (Iida, 2023). This may lead to a constrained sense of self, which is consistent with collectivist values.

Indigenous Chinese psychology highlights the concept of collectivism by demonstrating that core cultural constructs such as harmony, filial piety, and parental obligation are deeply embedded in individuals’ psychological orientations, thereby framing the self as fundamentally relational and interdependent rather than autonomous (Bedford & Yeh, 2019; Pan et al., 2013; Wu & Chao, 2017; Yuan et al., 2024). Professor Kuo-Shu Yang (1999) argued that indigenous Chinese psychology, as a culturally grounded emic approach, is better suited than Western-centric psychology for examining social phenomena in the Chinese context. Furthermore, Yang (2006) introduced the conservative values of “social orientation” as a conceptual system that emphasizes interdependence, relational roles, and social obligation. In contrast, the concept of “individual orientation” reflects modern values centered on autonomy, personal agency, and self-expression. The coexistence of internalized traditional values and contemporary values in the participants’ generation may lead to the subordination of personal awareness to social expectations.

### Locus of Control

Rotter (1954) argued that individual behaviour is shaped by learning experience, social context and expectations. Consequently, behavioural tendencies are considered variables that contribute to the formation of personality traits. He later proposed the concept of locus of control, suggesting that analysing the nature of personal beliefs can help predict individuals’ behavioural tendencies. This theoretical construct introduced a conceptual motivational framework for behavioural control, laying the foundation for key theoretical developments in human behaviour, learning and mental health, most notably Seligman’s theory of learned helplessness (Seligman, 1975). Wallston et al. (1978) developed the Multidimensional Health Locus of Control Scale, which was based on the theory of locus of control and specifically designed to identify key motivational indicators underlying individuals’ adherence to medical recommendations and engagement in health-promoting behaviours. Building on this research, subsequent studies have used assessment results to inform psychological interventions aimed at enhancing perceived control over life events, thereby improving mental health (Blair et al., 1999). In summary, locus of control is conceptualised as a personal trait that can serve as a foundational belief system to enhance individuals’ performance in health-related behaviours. As a personal trait, internal locus of control reflects the belief that outcomes stem from one’s own efforts and decisions. Individuals with this orientation tend to exhibit stronger learning transfer, greater self-regulation and persistence, even in the face of setbacks. By contrast, an external locus of control is characterised by attributing outcomes to external forces such as luck or situational factors, which may hinder experiential learning and limit strategic behavioural adjustment. In the long term, individuals with an external locus of control are more prone to feelings of helplessness and tend to lack initiative and persistence. Their behaviours are often contingent upon external rewards and punishments, with limited support from intrinsic motivation (Rotter, 1966). Interestingly, one participant exhibited an external locus of control, in contrast to the others, which may be better understood through the lens of indigenous Chinese psychology.

In Eastern societies, the examination of psychosocial phenomena calls for the integration of culturally embedded philosophical wisdom, such as Confucianism, Buddhism and Daoism. Within these ideologies, the concept of *yuanfen* (緣份) is deeply embedded in Chinese belief systems and is commonly recognised by individuals in Chinese cultural communities (Hsu & Hwang, 2016). Based on Hsu and Hwang’s (2016) theoretical model of *Yuanfen*, this study conceptualises a culturally grounded psychological adjustment process in Chinese societies, which is divided into two components: *yuan* (predestined affinity) and *fen* (actualization or deserved share). Individuals who cognitively engage more with the former tend to hold views aligned with an external locus of control, whereas those who engage more with the latter exhibit tendencies similar to an internal locus of control. By contrast, individuals who fail to activate either schema may be more vulnerable to experiencing negative emotions. Thus, the authors argued that either pathway represents a culturally transmitted coping mechanism that facilitates internal psychological adjustment. The authors emphasise that internal processes are not necessarily limited to dichotomous choices. Instead, individuals often navigate the grey area between extremes to identify the most appropriate course of action in response to external dissatisfaction. This dialectical belief of *yuanfen* contrasts with the Western tendency to adopt a clear stance as a representation of personal values or behavioural orientation.

### Self-Determination and General Skills

Although three participants recalled encountering minor challenges since joining the programme years ago, they consistently demonstrated positive psychological dispositions and adopted proactive strategies to sustain participation in health-related benefits. Their goal-directed mindset reflects the core components of Snyder’s Hope Theory, which conceptualises human agency and pathways thinking as key cognitive-motivational elements that facilitate adaptive coping and resilience in the face of adversity (Snyder, 2002; Snyder et al., 2000). Participants showed a strong orientation towards both agency and pathways, enabling them to persist despite age-related setbacks and physical limitations. Moreover, they maintained a positive outlook and continued social engagement, even when their physical performance fell short of ideal standards. These findings align with Rowe and Kahn’s (1987) conceptual framework of successful ageing, which includes three essential components: low risk of disease and disability, high cognitive and physical functioning, and active engagement with life.

### Self-Efficacy

All participants in this study reported positive experiences and expressed coherent beliefs, and these findings were in line with previous research. Extensive research has consistently emphasised the critical role of self-efficacy in initiating and sustaining health-related behaviours. Specifically, among adults aged 60 years and above, a strong sense of self-efficacy has been significantly associated with continued engagement in health-related behaviours (Silva & Lautert, 2010). Self-efficacy has been identified as a potential mechanism for mitigating health risk factors. Previous study involving adults aged 55 and older found significant associations between self-efficacy and multiple behavioural factors, underscoring its potential value as a key factor in shaping health behaviour interventions (Dzerounian et al., 2022). Offering a meaningful contribution to the existing literature, this study interpreted older adults’ personal experiences and perspectives on self-efficacy in sustained participation in regular group exercise from a qualitative research perspective.

While the analysis focused primarily on personal determinants, the findings also revealed broader social and environmental influences. In terms of self-efficacy, participants’ beliefs were reinforced not only by personal convictions but also through their engagement with broader contexts. Socially, participants emphasized that maintaining their health through exercise was a way to avoid becoming a burden on their children, a concern deeply rooted in the tradition of filial piety. Environmentally, participants’ efficacy beliefs were informed by their awareness of the high prevalence of bedridden older adults in Taiwan. This collective experience underscored the consequences of physical decline and reinforced their determination to sustain exercise as a preventive strategy.

### Implications for Health Promotion Practice

Based on the perspectives identified in the findings, this study offers practical recommendations for health promotion practitioners to reduce potential barriers to participation in group-based exercise programmes. When designing activities or interacting with participants, practitioners should avoid assuming that all individuals possess extroverted or outgoing dispositions. Instead, it is important to consider diverse personality traits and emotional sensitivity. For example, the concerns of more introverted individuals should be proactively addressed, and the relational needs of more emotionally sensitive participants should be met through appropriate interpersonal strategies. In addition, exercise facilitators should provide more guidance to help participants recognise their progress throughout the process. By highlighting incremental improvements, practitioners can foster a positive feedback loop that enhances participants’ self-efficacy, thereby encouraging sustained engagement. Rather than focusing exclusively on immediate performance outcomes, emphasising reflective practice that centres on small-scale personal accomplishments may reduce negative self-perceptions and prevent discouragement, particularly among individuals who feel their abilities fall short of expectations.

### Implications for Inactive Older Adults

From a psychological perspective, ageing should be understood not as a process of decline but as a meaningful developmental stage. For midlife adults without regular exercise habits, embracing this view may help reframe the present as a phase of renewal rather than a limitation. Life transitions call for intentional reflection. This begins with a respectful farewell to one’s former self, followed by a transitional period in which time and self-reflection are essential for internal adjustment. In time, this process cultivates an openness to new possibilities, and attending to inner signals can help individuals discern their personal growth throughout the transition.

A well-developed capacity for self-adjustment enables individuals to accept their current selves, gain clarity on life goals, and experience a deeper sense of fulfilment. In this study, participants demonstrated psychological adaptability by acknowledging their physical limitations without internalising self-critical thoughts. Rather than feeling discouraged by unmet performance expectations, they embraced their present capabilities and sustained participation with the long-term aim of maintaining their quality of life. By sharing these perspectives, this study seeks to inspire older adults who have yet to develop regular exercise habits to initiate physical activity and, through that journey, foster greater self-awareness.

## Conclusion

This study explored how older adults sustained long-term participation in group-based exercise programmes through the lens of the personal stream of the TTI. The findings revealed that personality traits such as conscientiousness and agreeableness were consistently evident, while openness, extraversion, and neuroticism appeared more selectively across individuals. A composed sense of self characterised most participants, whereas a constrained self-concept reflected the influence of familial expectations. Locus of control highlighted the interplay between personal agency and external reliance. Self-determination and general skills were expressed through persistent effort and self-directed practice. Finally, self-efficacy was reinforced through perceived accomplishment, encouragement, and vicarious experiences. Collectively, these determinants shaped psychological scripts that reinforced long-term participation.

Taken together, the results underscore the central role of self-determination, general skills, and self-efficacy in facilitating perseverance and sustained engagement. These findings not only advance understanding within the TTI framework but also offer practical guidance for designing exercise programmes that foster sustained participation among ageing populations.

### Avenues for Future Research

Although the TTI theoretical framework has been widely applied in quantitative research, few studies have explored it through qualitative inquiry. Given that this qualitative study focused on the personal stream of TTI, future research may extend this approach to enhance the comprehensiveness of the theory’s application. In addition, this study focused exclusively on highly active older adults who had sustained participation in the programme for at least five years. While this design provided insights into the determinants that support long-term engagement, it did not capture the perspectives of individuals who are less active or who do not participate at all. Future studies could address this by recruiting participants with varying levels of activity, thereby enabling comparisons that may reveal both barriers to participation and facilitators of sustained involvement. Moreover, longitudinal or mixed-methods designs would enable researchers to examine how participants’ experiences evolve over time and to better capture potential causal processes.

### Limitations

In order to contextualise the analytical approach adopted in this study, the present work draws upon the TTI, a comprehensive theoretical framework that synthesises multiple micro-level theories to elucidate the principal determinants of individual behaviour, organised according to their relative influence. Rooted in an extensive body of scholarly literature, this theory provides a robust conceptual foundation for understanding health behaviours. In this study, the selected determinants were carefully defined and interpreted in accordance with their theoretical origins to ensure conceptual rigour and analytical precision. Nevertheless, while this theoretical grounding offers structural coherence, the focus on six core constructs may constitute a limitation. It may have inadvertently constrained a more holistic understanding of each determinant’s complexity and multidimensional nature. Accordingly, the findings ought to be interpreted with an appreciation that the broader conceptual richness of each construct may not have been fully captured.

In addition, this study was limited by its small sample size of five participants. However, such a sample size has also been observed in other qualitative gerontological inquiries that prioritize depth over breadth. For instance, Sadang et al. (2021) conducted an instrumental case study with five older adults to explore adaptation strategies during the COVID-19 pandemic. Although the small number restricts the transferability of the findings to broader populations, each participant had been continuously enrolled in the programme for at least five years, which allowed for the collection of rich and detailed narratives on sustained engagement. The findings should therefore be interpreted as in-depth insights derived from the lived experiences of a small group of highly active older adults, rather than as universally generalizable claims.

## Data Availability

The data supporting the findings of this study are available from the corresponding author upon reasonable request.

## Appendix

Guiding questions:

Section 1: Background information.

Could you begin by describing your family background and upbringing?

Section 2: Personality traits (openness, conscientiousness, extraversion, agreeableness and neuroticism).

In what ways do you think your personality has been shaped throughout your life experiences?

- In your daily life or work, do you often apply new perspectives, imagination, or creativity?
- Do you usually plan or schedule tasks in advance? When managing responsibilities, do you generally proceed according to plan?
- Do you enjoy participating in large social activities or gatherings? How do you usually engage with others in these contexts, and what kinds of roles do you tend to take on?
- How do you typically engage with others in your daily interactions? How do you experience and respond when others negative emotions toward you?
- How do you perceive your emotional sensitivity? Do you feel your feelings are easily triggered by external events?

Section 3: Sense of self.

How well do you feel you understand your own emotional experiences? What strategies or practices do you use to increase self-awareness? How do you manage your responses when faced with others’ evaluations?

Section 4: Locus of control.

How do you usually understand the outcomes of the tasks you take on? Do you see them as more connected to your own efforts or to external factors?

Section 5: Self-determination and general skills.

During your participation in group exercise, what kinds of challenges or obstacles have you encountered? How did you adjust your mindset in response? How did you go about overcoming these difficulties?

Section 6: Self efficacy.

Looking ahead, how confident are you in continuing to participate in group exercise? How might this sense of capability influence other areas of your life?
